# Exposure to air pollution and self-reported effects on Chinese students: A case study of 13 megacities

**DOI:** 10.1371/journal.pone.0194364

**Published:** 2018-03-16

**Authors:** Sohail Ahmed Rajper, Sana Ullah, Zhongqiu Li

**Affiliations:** School of Life Sciences, Nanjing University, Nanjing, Jiangsu, P.R. China; The Ohio State University, UNITED STATES

## Abstract

Air pollution causes severe physical and psychological health complications. Considering China’s continuously-deteriorating air quality, this study aimed to assess the self-reported effects of air pollution on the behavior and physical health of the students of 13 densely populated cities, and their awareness, practices, and perception of air pollution and its associated public health risks. A detailed, closed-ended questionnaire was administered to 2100 students from 54 universities and schools across China. The questionnaire, which had 24 questions, was categorized into four sections. The first two sections were focused on air pollution-associated behavior and psychology, and physical effects; while the final two sections focused on the subjects’ awareness and perceptions, and practices and concerns about air pollution. The respondents reported that long-term exposure to air pollution had significantly affected their psychology and behavior, as well as their physical health. The respondents were aware of the different adverse impacts of air pollution (respiratory infections, allergies, and cardiovascular problems), and hence had adopted different preventive measures, such as the use of respiratory masks and glasses or goggles, regularly drinking water, and consuming rich foods. It was concluded that air pollution and haze had negative physical and psychological effects on the respondents, which led to severe changes in behavior. Proper management, future planning, and implementing strict environmental laws are suggested before this problem worsens and becomes life-threatening.

## Introduction

Air pollution is of serious concern across the globe, and is fueled by rapid population growth, continuous urbanization, increases in industrialization, continuous rises in energy demand, deforestation, and increases in car density, especially in major cities [1, 2]. Various anthropogenic activities lead to atmospheric degradation, such as emissions from vehicles, especially those that are older or poorly maintained; coal-powered industrial activities; construction, which produces dust; foundries and smelters; tobacco use; combustion that produces enormous heat; metal-based industries; mining; and excessive pesticide and chemical use [3–7]. This bleak scenario is further worsened through poor environmental management and regulation, use of inefficient technologies (with low production and high environmental deterioration), construction of congested roads, and the inability to strictly implement environmental regulations and laws, as well as a lack of awareness among the population about the serious health and psychological outcomes of pollution. This issue is even more prominent in underdeveloped and developing countries, where it is a serious concern as it adversely affects public health, alters the quality of life, and impacts the economy (by affecting agricultural production, for example) [8, 9].

Atmospheric heavy metals (lead, mercury, copper, zinc, cobalt, nickel, and cadmium), SO_2_, CO, NO_2_, benzene, particulate matter (PM), polycyclic aromatic hydrocarbons (PAHs), and chlorofluorocarbons (CFCs), are the primary contributors to air pollution [7, 10–12]. The presence of these pollutants at levels beyond permissible limits causes serious health issues, such as breathing problems, allergies, cancers, cardiovascular and respiratory diseases, and even mortality [12, 13]. Elders, infants, toddlers, children, sensitive people, and those suffering from asthma and such other disorders are more vulnerable (physically and psychologically) to the effects of air pollution [14, 15]. Higher temperatures and air pollution are also associated with low mood and potency [16, 17], changes in sexual behavior, and negative effects on reproductive health [17, 18]. The World Bank [19] has provided an excellent updated insight of the fertility rate for all countries around the world, based on total births per woman, which could be linked to global warming/greenhouse gases and air pollution [17, 20, 21]. Kihal-Talantikite et al. [22] extensively reviewed the adverse impacts of proximity to polluted areas on the outcomes of pregnancy, such as infant mortality, premature birth, low birth weight, congenital malformation, intrauterine growth retardation, and gestational age. The physical effects associated with air pollution are widely studied, reported, and reviewed [23–28].

Numerous studies have demonstrated a positive correlation between air pollution and mortality across the globe, including in China [12, 29–32]. Researchers have reported that 3–7 million deaths over the past few decades were caused by cardiovascular diseases (CVDs) after continuous exposure to excessive levels of airborne PM [33–36]. Industrial power plants, vehicle emissions, fossil fuels, and biomass combustion have been identified as major sources of PM, which acts as a primary pollutant and a secondary product of different gases, including NH_3_, NO_x_, and SO_2_ [12, 27, 34, 35]. Time-series studies have assessed the association between daily mortality and ambient air pollution in different western countries [29, 37–39], and different major Chinese cities, including Hong Kong [40], Wuhan [31], Guangzhou [41], Shanghai [30], and Beijing [27].

Approximately 800 million people were affected by the hazardous dense haze (covering an area of 1.4 million km^2^) in January 2013, which generated serious concern about air pollution among the residents of Eastern China [15, 42]. Yang et al. [32] reported that 1.2–2 million deaths per annum in China are attributed to air pollution, which was the fourth most prominent cause of disability-adjusted lifespans. Lu et al. [43] reported that rapid industrial, urbanization, and population growth are major sources of China’s continuously-deteriorating air quality, and are posing a significant threat to public health. Approximately 65–75% of industrial powerhouses in China are still coal-operated (i.e., coal is a major fuel source), and are designated as major pollution sources [6, 44]. Owing to the current situation, air reporting systems have been developed and installed in 190 cities (945 sites) across China. This system reports air quality on an hourly basis, focusing on six air pollutants: PM_2.5_ (PM<2.5 μm), PM_10_ (PM<10 μm), SO_2_, NO_2_, O_3_, and CO. Estimating and understanding outdoor air pollutants and their burden on public health would help to control air pollution [13, 43].

Air pollution is one of the leading factors that upsets human emotions and alters behavior [45, 46]. Long-term exposure to polluted air results in a variety of psychological problems (such as stress, depression, anxiety, irritation, becoming short-tempered, and mood swings), which adversely affect behavior (such as eating, recreation, commuting, traveling, and socialization) [13, 23, 47, 48]. A recent study indicated that older people and females were suffered more and were more anxious because of low air quality than younger people and males [49]. Various studies have demonstrated the positive impacts of clean air on the human psyche, resulting in a pleasant mood and positive behavior [50]. Similarly, different studies have revealed a positive correlation between increased criminality (aggressive and violent behaviors) in humans and elevated temperatures (specifically in the summer) and air pollution [51–53]. Air pollution has also been correlated with depression, a serious mental disorder affecting people globally, which is continuously increasing [54]. Depression is characterized by a loss of pleasure and interests, guilt, sadness, inter alia, decrease in libido, disruption to sleep, and a loss of concentration [17]. There are several studies available that show a positive correlation between air pollution and depressive disorders that adversely affect human behavior [55–57].

Air pollution-associated mortalities [27, 32]; and age-, gender-, and season-dependent adverse impacts/effects of air pollution on humans [58–60] are well documented, but most of these studies focused on physical factors. Therefore, an effort was made to study the effects of air pollution on both the physical and psychological health of Chinese students using a detailed questionnaire, and the first two portions were dedicated to each physical and psychological health.

## Material and methods

The study was conducted according to the Ethics Review Committee of Nanjing University (No. 2009–116). The Questionnaires were administered to the students following informed verbal consent. All participants were thoroughly informed of the purposes and contents of the project. All participants had the right to answer all or part of the questionnaire, and the right to stop participating at any time-point. They were requested to complete the questionnaires, then and there. Using questionnaire for data/information acquisition, the committee approves informed verbal consent, and hence it was considered to lessen time spent by the students or/and trouble to the students.

**Fig 1:**
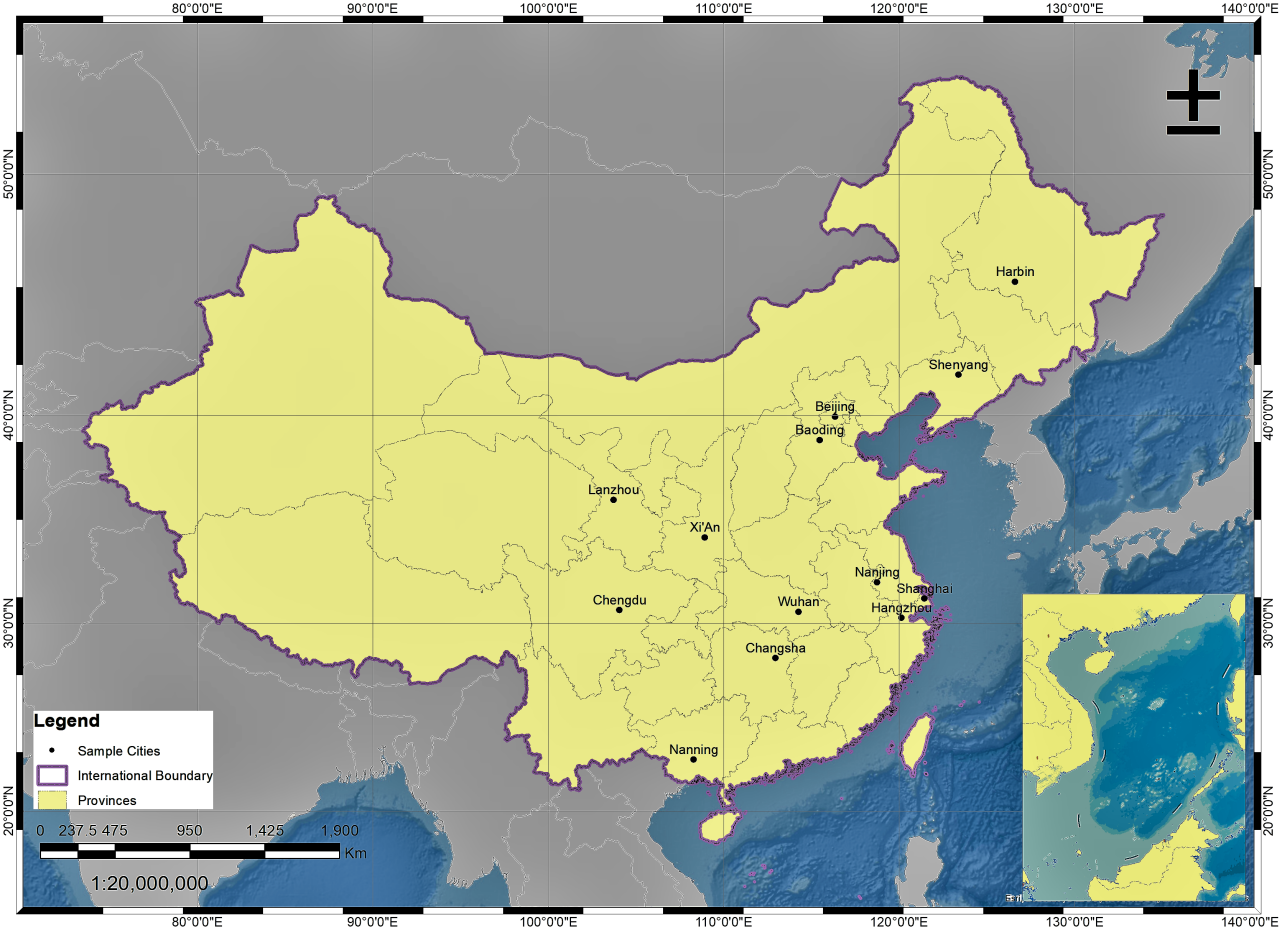
Geographical locations of the sampled cities.

In this study, a comprehensive questionnaire (S1 File) was distributed to 2100 randomly-selected students from the chosen universities and schools. Questionnaires that shared identical answers to all questions, and those with a large number of missing answers, were excluded from the analysis. After removing these questionnaires, a total of 2048 questionnaires from 13 major cities across China were analyzed, including Beijing (Northern China), Baoding (Northern China), Nanjing (Eastern China), Shanghai (Eastern China), Hangzhou (Eastern China), Xi’An (Northwestern China), Lanzhou (Northwestern China), Harbin (Northeastern China), Shenyang (Northeastern China), Chengdu (Southwestern China), Nanning (Southern China), Changsha (Central China), and Wuhan (Central China). The sampled cities are indicated in Fig 1.

The questionnaire contained a series of questions to elucidate the individual impacts of haze and air pollution on the physical and psychological (behavioral) health of the subjects. The questionnaire was prepared in two languages, English and Chinese. The questionnaire was split into four sections, two of which were dedicated to the effects of air pollution on behavior (caused by psychological adversity) and physical human health, while the final two assessed the respondents’ knowledge, attitude, and practices (KAP); and sources of knowledge and perception of air pollution and its associated health risks. The survey was conducted over a period of six months, from October 2015 through March 2016. This period was selected because air pollution and haze are often at their highest during these six months.

Haze appears for ≥100 days per year in the Yangtze River Delta, and for ≥200 days in some of the studied Eastern China cities [25, 61, 62]. The surveyed cities were chosen as they are more densely populated (megacities) than other smaller cities. The primary data was obtained through the questionnaire that was administered to students of 54 different universities and schools, which had lived there for over a year, across the sampled locations. The subjects were approached at different points of the universities and schools, including campus areas, playgrounds, laboratories, cafeterias, classrooms, libraries, coffee shops, and waiting rooms, to ensure that individuals with different mood states at that time were included, so that the broader impact of pollution on them could be observed. Similarly, it was ensured that universities and schools situated near roadsides (transportation emissions), in heavily congested and industrialized areas (high levels of combustion, effluent emissions, and fossil fuel use), as well as those in less crowded areas, were included.

The data obtained were imported and analyzed by MS Excel, Origin Pro8.5, and Statistix (Version X) respectively. Based on the previous studies, a Chi-Square (independence) test was conducted to determine the association of gender and age with the responses to all sections of the questionnaire and examine gender- and age-dependent effects, perceptions, attitudes, and practices. Descriptive statistics (frequencies/proportion) were used to summarize the demographics of the respondents, their attitudes, practices, and perceptions to air pollution and its associated health risks, as well as the adverse effects of air pollution on their behavior (psychology) and physical health. A *p*-value below 0.05 (two-sided) was considered to be statistically significant. To avoid family-wise error, Bonferroni type adjustment (Bonferroni correction) was carried out by adjusting α value (α_original_), by defining new α value (α_altered_ = 0.05/number of possible comparisons/analysis for each of the question, shown in Table A in S2 File). The map presenting the sampled cities was prepared in ArcGIS (V. 9.3).

## Results

The study included a total of 2048 subjects, who were recruited from 54 universities and schools across China. The largest number of individuals was recruited from Nanjing (21.2%), followed by Beijing (12.5%) and Changsha (11.2%). It was ensured that participants from different age groups were included. Of the total respondents, 47.3% were 16–25 years old. The study considered both genders; 55.2% of the subjects were female, while 44.8% were male. Table 1 presents the demographics of the respondents that took part in this study. Table 2 shows the average of air quality pollutants for the sampled cities. Table B in S2 File shows the interquartile ranges for the air pollutants across the samples cities (Oct 2015—March 2016).

**Table 1:** Demographics of the respondents across sampling sites/cities.

Cities	Universities/College	Gender			Age (Years)				
		Male	Female	Total	16–20	21–25	26–30	31–35	≥ 36
Nanjing	10 (18.5)*	178 (40.8)	258 (59.1)	436 (21.2)	232 (23.9)	150 (15.4)	38 (46.9)	9 (60)	7 (50)
Shanghai	5 (9.2)	62 (36.9)	106 (63.1)	168 (8.2)	32 (3.3)	135 (13.9)	1 (1.2)	0	0
Beijing	10 (18.5)	125 (49.1)	130 (50.9)	255 (12.5)	193 (19.9)	60 (6.1)	1 (1.2)	1 (6.7)	0
Hangzhou	3 (5.5)	52 (42.9)	69 (57.0)	121 (5.9)	79 (8.1)	39 (4.1)	3 (3.7)	0	0
Xi’An	5 (9.25)	94 (50.6)	88 (48.3)	182 (8.8)	40 (4.1)	133 (13.7)	9 (11.1)	0	0
Chengdu	2 (3.7)	27 (35.5)	49 (64.5)	76 (3.7)	7 (0.7)	66 (6.8)	3 (3.7)	0	0
Baoding	4 (7.4)	65 (43.9)	83 (56.1)	148 (7.2)	78 (8.1)	70 (7.2)	0	0	0
Nanning	4 (7.4)	47 (44.3)	59 (55.6)	106 (5.1)	32 (3.2)	73(7.5)	0	0	1 (7.1)
Harbin	2 (3.7)	42 (48.8)	44 (51.2)	86 (4.2)	78 (8.1)	8 (0.8)	0	0	0
Lanzhou	2 (3.7)	52 (42.9)	69 (57.1)	121 (5.9)	83 (8.5)	38 (3.9)	0	0	0
Changsha	4 (7.4)	132 (57.6)	97 (42.3)	229 (11.2)	62 (6.4)	149 (15.4)	13 (16.1)	3 (20)	2 (14.3)
Shenyang	1 (1.9)	0	30 (100)	30 (1.5)	0	18 (1.8)	12 (14.1)	0	0
Wuhan	2 (3.7)	42 (46.7)	48 (53.3)	90 (4.4)	54 (5.6)	29 (2.9)	1 (1.2)	2 (13.3)	4 (28.6)
Total	54 (100)	918 (44.8)	1130 (55.2)	2048 (100)	970 (47.4)	968 (47.3)	81 (3.9)	15 (0.7)	14 (0.7)

*Percentage given

**Table 2:** Air pollution across the sampled cities (Oct 2015–March 2016) (Mean±SD).

Cities	Seasons	AQI	PM 2.5 (μg/m^3^)	PM 10 (μg/m^3^)	SO_2_ (μg/m^3^)	NO_2_ (μg/m^3^)	CO (mg/m^3^)	O_3_ (μg/m^3^)
Nanjing	Autumn*	98.6±26.3	68.7±22.0	113.1±28.1	20.5±4.1	58.9±6.3	1.1±0.1	43.6±20.8
	Winter**	106.3±5.7	74.5±5.3	123.0±8.7	22.1±2.5	53.3±6.2	1.3±0.1	51.9±12.6
Shanghai	Autumn	89.2±19.7	62.6±18.1	79.7±18.4	18.8±5.1	56.7±9.4	1.0±0.2	53.3±28.5
	Winter	84.5±10.9	58.6±9.5	78.8±5.3	18.9±3.6	48.7±7.1	0.9±0.2	65.5±14.5
Beijing	Autumn	141.1±39.8	111.0±38.4	107.0±36.9	11.4±6.6	61.0±11.3	1.9±0.9	23.2±13.6
	Winter	96.9±32.6	66.1±24.4	90.4±38.1	16.5±2.6	45.5±11.5	1.2±0.3	41.7±10.9
Hangzhou	Autumn	87.1±26.5	62.5±21.3	93.9±29.2	15.9±2.6	53.6±6.3	1.0±0.2	36.8±22.9
	Winter	94.7±8.1	67.7±7.3	107.9±10.8	14.2±1.3	49.8±11.0	0.9±0.1	48.9±16.8
Xi’An	Autumn	115.0±32.0	75.8±28.4	160.7±44.7	29.4±12.7	51.4±7.6	2.1±0.7	20.4±7.0
	Winter	131.1±21.2	86.2±23.3	180.0±30.1	31.5±7.0	53.9±8.7	2.3±0.7	31.7±8.3
Chengdu	Autumn	88.5±22.6	63.4±18.9	104.7±27.7	16.0±1.3	53.0±8.2	1.1±0.2	26.1±13.7
	Winter	99.4±6.2	70.4±6.7	118.4±10.0	15.4±2.0	54.1±6.7	1.3±0.0	41.5±11.7
Baoding	Autumn	168.9±75.7	134.6±69.9	195.5±83.7	60.4±32.4	78.1±18.6	2.7±1.8	26.4±14.7
	Winter	139.4±40.9	102.2±40.2	159.9±45.0	68.4±15.7	69.2±14.3	2.4±1.0	36.7±16.5
Nanning	Autumn	64.05±13.6	41.23±9.6	74.92±24.9	14.15±2.4	35.28±3.5	1.03±0.01	37.07±12.7
	Winter	69.9±10.1	48.6±9.2	76.0±9.4	12.9±2.0	34.3±3.5	1.1±0.1	35.9±10.0
Harbin	Autumn	144.1±55.9	115.6±53.5	155.8±60.2	40.5±25.7	58.1±17.7	1.4±0.3	25.9±10.0
	Winter	98.9±22.6	70.6±20.8	99.7±15.9	52.1±18.0	52.4±7.9	1.4±0.1	32.0±9.5
Lanzhou	Autumn	90.6±14.4	57.5±17.5	116.7±16.5	25.3±9.0	55.7±10.8	1.7±0.6	30.9±8.2
	Winter	101.8±12.3	55.2±8.5	136.4±22.6	23.7±10.4	49.0±7.2	1.5±0.4	48.6±13.8
Changsha	Autumn	86.3±23.2	61.6±17.3	83.3±23.5	14.5±2.2	41.5±2.7	1.0±0.1	37.3±27.8
	Winter	101.0±8.3	72.7±7.1	92.9±3.3	17.7±1.3	40.5±6.6	1.1±0.1	40.7±14.6
Shenyang	Autumn	131.5±33.0	104.2±37.4	140.7±36.7	75.3±45.6	51.4±5.6	1.2±0.3	34.4±10.9
	Winter	98.6±16.5	63.9±11.0	116.4±26.8	78.5±24.4	41.7±5.0	1.0±0.1	47.5±17.5
Wuhan	Autumn	107.6±32.8	77.8±25.7	111.4±32.9	19.8±2.1	51.5±8.7	1.2±0.1	36.4±23.8
	Winter	123.2±15.6	89.0±13.8	123.2±7.3	18.7±1.1	47.8±5.9	1.1±0.2	44.5±15.9

Autumn* = October to December 2015; winter** = January to March 2016

The first section of the questionnaire covered the adverse impacts of air pollution on the physical health of the recruited subjects. Of the total respondents, 88.9% reported that they have felt (always, often, and some of the time) the ill effects of air pollution, suggesting that air pollution is a major public health concern across China. The subjects suffered from different effects at varying magnitudes; 25.0% often suffered from sneezing, a dry throat, and eye irritation, and 34.0% occasionally experiences these effects. Of the total respondents, 62.0% had suffered (always, often, and sometimes) breathing problems and have faced respiratory problems. Fewer people suffered from coughing/wheezing and headaches/dizziness than those that have suffered from other ENT (ear, nose, and throat) problems. In total, 9.1%, 10.5%, and 24.6% of the respondents always, often, and sometimes felt that they had lower energy levels in response to air pollution, respectively. For sleeping patterns, 8.7%, 11.5%, and 23.4% of the respondents always, often, and sometimes faced problems in their sleeping patterns, respectively. Table 3 shows the reported physical health effects caused by air pollution, while Tables 4 and 5 contain gender- and city-, and age-dependent responses, respectively.

**Table 3:** Air pollution/haze caused physical health effects reported by the respondents.

Physical effects	Always (%)	Often (%)	Sometimes (%)	Rarely (%)	Never (%)
Felt air pollution effects	622 (30.4)	580 (28.4)	618 (30.1)	201 (9.8)	27 (1.3)
ENT problems/irritation/allergies	333 (16.3)	510 (24.9)	693 (33.8)	414 (20.2)	98 (4.8)
Respiratory problems	241 (11.8)	401 (19.6)	627 (30.6)	533 (26.0)	246 (12.1)
Coughing or wheezing	210 (10.2)	390 (19.1)	568 (27.7)	604 (29.5)	276 (13.4)
Headaches and dizziness	174 (8.5)	295 (14.4)	567 (27.7)	629 (30.7)	383 (18.7)
Reduced energy level	186 (9.1)	217 (10.5)	503 (24.6)	632 (30.8)	510 (24.9)
Sleeping disorder i.e., insomnia	179 (8.7)	236 (11.5)	479 (23.4)	596 (29.1)	558 (27.2)

**Table 4:** Gender & city-dependent physical effects of air pollution on the respondents.

Gender	Always		Often		Sometimes		Rarely		Never		*P*-value*	*χ*^*2*^
	*n*	%	*n*	%	*N*	%	*n*	%	*N*	%		
Effects of air pollution felt												
Male	287	14.0	259	12.6	271	13.2	88	4.3	13	0.6	0.926^b^	0.89
Female	335	16.4	321	15.7	347	16.9	113	5.5	14	0.7		
Sneezing, runny nose, dry throat, or eye Irritation												
Male	134	6.5	236	11.5	305	14.9	182	8.9	61	2.9	**0.003**^a^	15.60
Female	199	9.7	274	13.4	388	18.9	232	11.3	37	1.8		
Breath shortening or reduced lung function												
Male	116	5.4	189	9.2	256	12.5	243	11.7	119	5.8	0.109^b^	7.54
Female	124	6.3	212	10.4	371	18.1	290	14.2	126	6.2		
Coughing or wheezing												
Male	101	4.9	181	8.8	241	11.8	258	12.6	137	6.7	0.181^b^	6.24
Female	108	5.3	209	10.2	327	15.9	346	16.9	140	6.8		
Headache and dizziness												
Male	85	4.2	125	6.1	249	12.2	270	13.2	189	9.2	0.167^b^	6.46
Female	87	4.3	170	8.3	318	15.5	359	17.5	194	9.5		
Reduced energy level												
Male	92	4.5	103	5.0	232	11.3	246	12.0	245	11.9	**0.007**^b^	14.09
Female	92	4.6	114	5.6	271	13.2	386	18.8	264	12.9		
Sleep deprivation or sleeping disorders												
Male	95	4.6	113	5.5	214	10.4	240	11.7	256	12.5	**0.025**^b^	11.11
Female	84	4.2	123	6.0	265	12.9	356	17.4	301	14.7		
Adverse physical effects reported by the respondents across study sites/cities												
Nanjing	400	13.1	490	16.1	929	30.5	770	25.3	453	14.9	**0.000**^a^	368.18
Shanghai	226	19.2	167	14.2	313	26.6	272	23.1	198	16.8		
Beijing	229	13.4	337	19.7	419	24.4	472	27.5	258	15.1		
Hangzhou	67	7.9	132	15.6	247	29.2	240	28.3	161	19.0		
Xi’An	170	13.9	223	18.2	348	28.4	291	23.7	195	15.9		
Chengdu	44	8.3	92	17.3	175	32.9	158	29.7	63	11.8		
Baoding	219	21.1	242	23.4	291	28.1	197	19.0	87	8.4		
Nanning	55	7.3	205	27.3	217	28.9	156	20.7	119	15.8		
Harbin	111	18.4	126	20.9	143	23.8	149	24.8	73	12.1		
Lanzhou	81	9.6	162	19.1	268	31.6	204	24.1	132	15.6		
Changsha	160	9.9	292	18.2	470	29.3	452	28.2	229	14.3		
Shenyang	34	16.2	36	17.1	68	32.4	49	23.3	23	10.9		
Wuhan	79	12.5	125	19.8	167	26.5	178	28.3	81	12.9		
Total Responses	1875	13.2	2629	18.5	4055	28.5	3588	25.2	2072	14.6	14219	

* = bold value represents *p*-value < 0.05

^a^ = *p*-value < α_altered_ (Significant after Bonferroni correction);

^b^ = *p*-value > α_altered_ (Non-Significant after Bonferroni correction/adjustment)

**Table 5:** Age-dependent physical effects of air pollution on the respondents.

Age range	Always		Often		Sometimes		Rarely		Never		*P*-value*	*χ*^*2*^
	*n*	%	*n*	%	*N*	%	*n*	%	*n*	%		
Effects of air pollution felt												
16–20	293	29.9	262	26.8	300	30.7	107	10.9	16	1.6	0.577^b^	14.29
21–25	286	30.6	273	29.2	280	29.9	87	9.3	9	0.9		
26–30	33	31.7	34	32.7	29	27.9	6	5.8	2	1.9		
31–35	4	25.0	4	25.0	7	43.8	1	6.3	0	0		
≥ 36	6	40.0	7	46.7	2	13.3	0	0	0	0		
Sneezing, runny nose, dry throat, or eye Irritation												
16–20	168	17.2	237	24.2	314	32.1	217	22.2	42	4.3	0.681^b^	12.89
21–25	145	15.5	235	25.1	329	35.2	175	18.7	51	5.5		
26–30	17	16.3	28	26.9	38	36.5	17	16.3	4	3.8		
31–35	2	12.5	5	31.3	7	43.8	1	6.3	0	0		
≥ 36	1	6.7	5	33.3	5	33.3	4	26.7	1	6.7		
Breath shortening or reduced lung function												
16–20	130	13.3	190	19.4	275	28.1	265	27.1	118	12.1	0.395^b^	16.85
21–25	99	10.6	191	20.4	300	32.1	238	25.5	107	11.4		
26–30	10	9.6	17	16.3	39	37.5	24	23.01	14	13.5		
31–35	1	6.3	1	6.3	6	37.5	4	25.0	4	25.0		
≥ 36	2	13.3	2	13.3	7	46.7	2	13.3	2	13.3		
Coughing or wheezing												
16–20	111	11.3	189	19.3	260	26.6	283	28.9	135	13.8	0.675^b^	12.97
21–25	83	8.9	171	18.3	272	29.1	287	30.7	121	12.9		
26–30	12	11.5	24	23.1	29	27.9	25	24.0	14	13.5		
31–35	1	6.3	3	18.8	4	25.0	3	18.8	5	31.2		
≥ 36	1	6.7	3	20.0	3	20.0	6	40.0	2	3.3		
Headache and dizziness												
16–20	92	9.4	141	14.4	266	27.2	292	29.9	187	19.1	0.952^b^	7.87
21–25	70	7.5	137	14.7	260	27.8	298	31.9	169	18.1		
26–30	10	9.6	13	12.5	31	29.8	31	29.8	19	18.3		
31–35	1	6.3	1	6.3	6	37.5	4	25.0	4	25.0		
≥ 36	0	0	3	20.0	4	26.7	4	26.7	4	26.7		
Reduced energy level												
16–20	97	9.9	95	9.7	220	22.5	303	30.9	263	26.9	0.097^b^	23.65
21–25	76	8.1	110	11.8	246	26.3	294	31.4	208	21.3		
26–30	13	12.5	8	7.7	30	28.8	27	25.9	26	25.0		
31–35	0	0	1	6.3	5	31.3	5	31.3	5	31.3		
≥ 36	0	0	3	20.0	2	13.3	3	20.0	7	46.7		
Sleep deprivation or sleeping disorders												
16–20	105	10.7	99	10.1	208	21.3	275	28.1	291	29.8	**0.013**^b^	31.12
21–25	62	6.6	125	13.3	243	25.9	273	29.2	232	24.8		
26–30	12	11.5	8	7.7	21	20.2	36	34.6	27	25.9		
31–35	1	6.3	2	12.5	3	18.8	5	31.3	5	31.3		
≥ 36	0	0	2	13.3	4	26.7	7	46.7	2	13.3		

* = bold value represents *p*-value < 0.05

^b^ = *p*-value > α_altered_ (Non-Significant after Bonferroni correction/adjustment)

The next section of the questionnaire was focused on the behavioral and psychological impacts of air pollution. In total, 62.1%, 78.1%, and 65.5% of the respondents suffered from depression/sadness/unpleasant moods, reduced exercise routines/jogging speed/jogging duration, and reduced walking speed, respectively, due to air pollution. A total of 1136 (55.4%) respondents reported that they feel anxiety and frustrated during hazy days. Of the total respondents, 44.1% reported that they become aggressive due to haze/air pollution, and a higher number reported that they exhibit more aggressive behaviors during warmer days (63.9%) than they do during colder days (27.6%). Table 6 shows the behavioral effects of air pollution noted by the respondents, while Tables C and D in S2 File show the reported gender- and city-dependent, and age-dependent effects, respectively.

**Table 6:** Behavioral and psychological effects caused by air pollution reported by the respondents.

Behavioral effects	Yes (%)	No (%)
Depressed	1278 (62.4)	770 (37.6)
Jog faster and for a short time	1600 (78.1)	448 (21.9)
Walk faster	1341 (65.5)	707 (34.5)
Anxiety	1136 (55.5)	912 (44.5)
Aggressiveness	904 (44.1)	1144 (55.9)
Aggressiveness during cold days	566 (27.6)	1482 (72.4)
Aggressiveness during hot days	1308 (63.9)	740 (36.1)

The third section of the questionnaire contained questions about the preventive measures adopted by the respondents. Of the total respondents, 68.2% reported that they use a mask to cover their noses and mouths, 22.0% used eyeglasses/goggles to protect their eyes, 63.2% drink more water to help them flush out toxins (absorbed through the lungs/skin), and 65.2% indicated that they have a rich diet (high levels of vitamins C and E, and Omega-3-Fatty acids) to improve their immune response. Table 7 shows the practices adopted by the respondents to prevent the adverse impacts of air pollution, while Tables E and F in S2 File show the gender- and city-, and age-dependent practices, respectively.

**Table 7:** Preventive measures adopted to prevent the ill effects of air pollution.

Preventive measures	Yes (%)	No (%)
Use of respiratory mask (i.e., N95 (blocks about 95% of particles))	1396 (68.2)	652 (31.8)
Wear eyeglasses or goggles	451 (22.0)	1597 (78.0)
Drink more water	1294 (63.2)	754 (36.8)
Build immunity with rich food	1335 (65.2)	713 (34.8)

**Fig 2:**
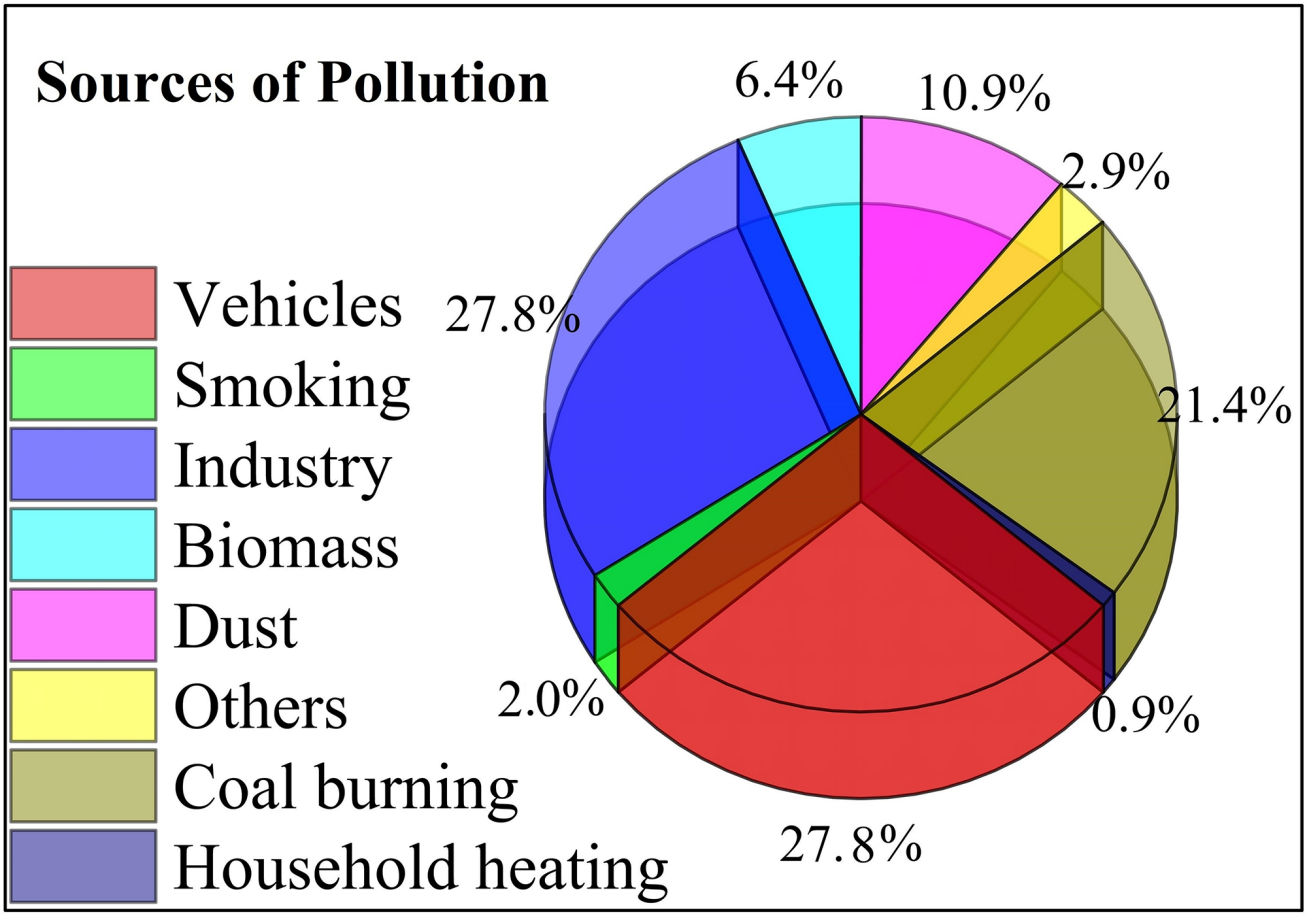
Sources of pollution reported by the respondents.

**Fig 3:**
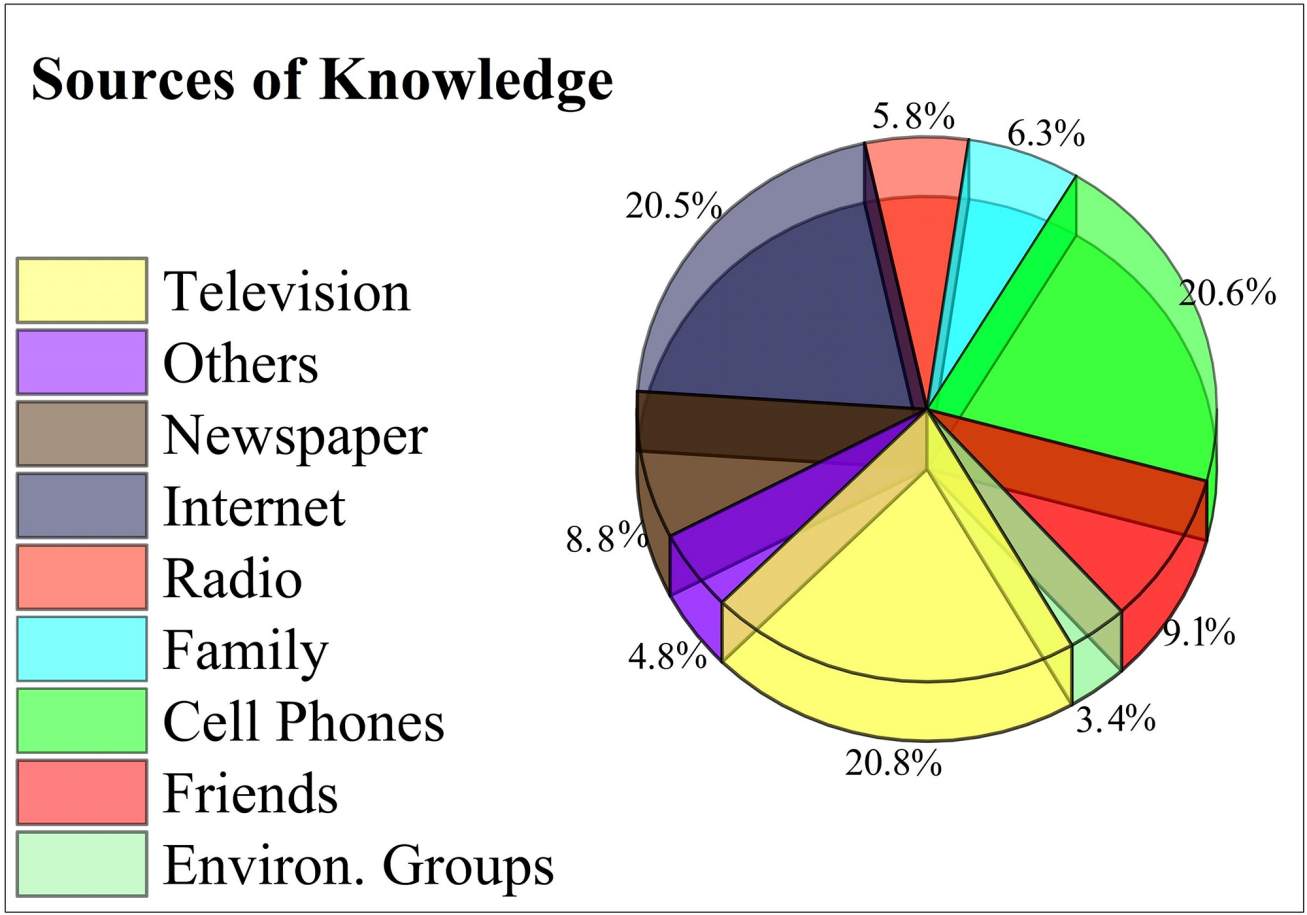
Sources of knowledge reported by the respondents.

The final section of the questionnaire evaluated the recruited individuals’ awareness levels and perception of pollution. Of the total subjects, 96.0% reported that smoking should be banned in public areas and restricted to designated zones, 69.3% were aware that air pollution causes cardiovascular diseases and respiratory problems, such as lung cancer, and that air pollution was one of the major causes of mortality in China over the last two decades. Regarding industrialization, 68.8% of the respondents did not agree with compromising their health because of environmental and air pollution originating from China’s development, economic strength, and GDP growth from industrialization. Of the total subjects, 91.9% were aware of major pollutants, such as CO, SO_2_, and NO_2_. Vehicle exhausts (27.8%), industrial emissions (27.8%), and coal burning (21.4%) were identified as major sources of air pollution (Fig 2). Television (20.8%), cell phones (20.6%), and the internet (20.5%) were the leading sources of information about air pollution and its adverse health effects (Fig 3). Table 8 shows the respondents’ levels of awareness and perception of air pollution and its associated health risks, while Tables G and H in S2 File show the gender- and city-, and age- dependent knowledge and perceptions of air pollution, respectively.

**Table 8:** Respondents’ levels of awareness and perceptions of air pollution.

Awareness/Perception	Yes (%)	No (%)
Smoking should be prohibited	1966 (96.0)	82 (4.0)
Air pollution associated deaths	1419 (69.3)	629 (30.7)
Accept health loss over GDP growth	640 (31.2)	1408 (68.8)
Awareness about air pollutants	1882 (91.9)	166 (8.1)

## Discussion

China is currently facing prominent and complex issue of air pollution due to a rapid increase in urbanization and economic growth caused by heavy industrialization [43, 63, 64]. Environmental degradation could become more severe if proper management, environmental safety, and planning are not assured by environmental protection agencies, and the current scenario continues [11]. Over the past two decades, the infrastructure of almost all major Chinese cities has improved considerably, with extensive reconstruction in urban areas and modernization of industries [49]. In China’s major cities, subway systems are under development, along with other governmental and non-governmental infrastructures. The construction and expansion of these other infrastructures have attracted large numbers of workers from the countryside, elevating the population density of these cities. They have also resulted in an increase in urban air pollution, increasing the vulnerability of residents to adverse health effects [11, 65]. The ill-effects of long-term exposure to air pollution on human health, such as weakening/damaging the immune system, respiratory problems, low birth weight, and increased head circumference of children at birth, have been well-document [66–73]. Air pollution can cause mortality if different pollutants are present and exceed their permissible limits, for example, from continuous exposure to air containing 0.001% CO [74]. According to a report by the World Health Organization, urban air pollution (indoor and outdoor) contributes to over 2 million premature deaths per year [75]. According to the Global Burden of Disease study [26], of the 15 countries, China ranked first for premature mortalities (over 1.3 million premature deaths), which were attributed to ambient air pollution, while the preventable death rate was higher for Chinese megacities, including Beijing, Shanghai, Chengdu, and Tianjin [76].

The aim of this study was to develop a more comprehensive model for the widely prevailing social problem of air pollution, which covered almost the whole of China, but specifically major cities. This study provides better insight for breaching the gap between public health KAP and scientific research than previous studies on this issue, as they were confined to certain places, such as a single city or province. However, our study was conducted in all of China’s major cities. This study strengthens the available literature [28, 49, 77] for all the relevant governing bodies, institutions, and agencies, and acts as a reference for mitigating the current scenario and preventing or reducing future risks and hazards caused by air pollution and its sources. According to Omanga et al. [78], an awareness of the public’s perception of pollution and its associated health risks is useful for designing and adapting suitable intervention programs.

Several studies have elucidated the impacts of air pollution on the respiratory system [68–70]. Respiratory disorders caused by air pollution include coughing, bronchitis, emphysema, and lung cancer [73], rendering individuals who are already suffering or have suffered from cardiac failure, chronic obstructive pulmonary disease, and asthma, more vulnerable [79]. According to Botkin and Keller [80], those already suffering from respiratory disorders are the most vulnerable to air pollution. In this study, over 98% respondents reported that they have felt adverse physical effects (ranging from experiencing them all the time to rarely) due to air pollution; while only 1.3% responded that they never felt such effects. This signified that the sampled cities currently have poor and polluted air conditions. Other reported physical problems including flu (a runny nose), a dry throat, eye irritation, shortness of breath, reduced lung functioning, coughing/wheezing, headache/dizziness, reduced energy levels, and sleeping disorders. The severity of these problems reported by the respondents was in various ranges. The variation in these responses could be attributed to the health status of the individuals, variations in the pollution levels of the studied cities, and genetic polymorphism [81–83]. The reported results are consistent with those of previous studies, revealing the same adverse health effects from exposure to air pollution [34, 84, 85].

Positive psychological effects, better mental health, and appropriate behavior result from a suitable and clean environment, and comfortable weather conditions [50, 86–88], while an unhealthy environment has adverse effects on behavior and psychological and mental health, and causes abnormalities [55–57, 89, 90]. Polluted air exacerbates stress and depression, and alters behavior [14, 56, 73]. Sahari et al. [91] reported that poor atmospheric conditions (air and temperature) around living spaces and residential areas was a major cause of stress in humans. According to Brealey [92], stress is contagious, and is a physical and emotional response to pressure. Shahrom [93] described stress as a feeling of discomfort in oneself that influences the mind, however, according to Fadzillah-Kamsah, stress covers the following feelings: burden, worry, strain, conflict, pressure, feeling, fatigue, helplessness, panic, and depression [94]. Sahari et al. [94] comprehensively reviewed stress, and Łopuszańska and Makara-Studzińska [21] provided an overview of air pollution-associated depression.

Different stressors that trigger behavioral changes in humans have been explored. Tay and Smith [95] identified that peoples’ working styles, job nature, recreation methods, lifestyle, and local weather affected their behavior. Turkington [96] listed financial condition, family problems, social life, home problems, illness, time mismanagement, and job/career problems as primary factors affecting behavior. Shahroom [93] suggested that spiritual, social, physical, and biophysical factors affect behavior, while the most recent research revealed that environmental conditions (including air pollution and temperature) are primary stressors, as indicated by a number of researchers [46, 55–57, 91, 94, 97–98]. Torres and Casey [48] identified that mental health, affected by climatic changes or air pollution, is one of the main reasons for migration and disruption of social ties.

Air pollution has an adverse effect on outdoor and sporting activities that are practiced for maintaining an individual’s health and fitness [99]. In this study, it was reported that 78.1% of the respondents jogged faster and for a shorter duration on hazy days, while 65.5% walked faster in response to air pollution. Air pollution change behaviors associated with sports, such as going outside for games, exercise, and gymnasia. The stamina and physical strength required for partaking in sports are reduced by air pollution, as pollutants such as heavy metals and PM penetrate the lungs deeply and are not easily removed by exhalation, altering the duration and general willingness of people to partake in such activities [65]. To remove toxic pollutants, 63.2% of the respondents reportedly drank more water on hazy days, compared to normal or pollution-free days. There was no significant difference in water intake between different age categories and genders, which demonstrated that all respondents drank enough water to minimize the risks of air pollution, irrespective of their age and gender. Awareness and knowledge of air pollution might improve this scenario, and could encourage normal sporting behavior and change the attitude of the population towards air pollution-associated adverse human behavior [100].

Previous studies [101, 102] reported that the perception and knowledge of air pollution and its associated health risk were significantly different between different genders and the health status of the recruited individuals. There was a significant (*p*<0.05) difference between male and female responses to smoke prevention in public areas and compromising on air pollution caused by China’s GDP growth, while there was no statistically significant difference between the genders’ awareness of air pollutants and their associated diseases/disorders. There was a significant difference between the subjects’ awareness based on age, excluding awareness of major air pollutants. Significant (*p*<0.05) differences were observed between the respondents in the sampled cities, for all the sections of the questionnaire, which could be attributed to the differences in the levels of air pollution, access to media, and the local/provincial government’s outreach activities, such as seminars, symposia, and campaigns, regarding air pollution and its associated health risks. The differences in air pollution across the sampled cities could be caused by whether the city is a zone of production or consumption. Production sites are directly associated with trade (export/import, local/international), which elevates air pollution, as indicated by Wang et al. [12] and Zhang et al. [103]. Variations in the pollution level across China’s provinces could be caused by the county’s size. There are considerable differences between provinces, such as energy endowments and resources, lifestyles, population densities, and economic development. It was recently observed that, due to these factors, pollutants emissions increased rapidly in central China, while they stabilized or even decreased in coastal China [104–106].

A linear relationship between the knowledge or perceptions of air pollution’s adverse health impacts and literacy level has been reported [107, 108]. Some studies also reported that there was a marked difference in the subjects’ knowledge and perception of air pollution between income classes [109, 110]. Liu et al. [49] and Zhang [111] reported that women are more worried towards air pollution in China than men. In this study, there was a significant difference (*p*<0.05) between the preventative measures adopted by male and female subjects; the female respondents were more cautious towards air pollution as they practiced more preventative measures, such as the use of respiratory masks, goggles/glasses, drinking more water, and consuming nutrient-rich meals to boost their immunity, as compared to males. Similarly, Kim et al. [63] reported that younger people were less satisfied with air quality than older people, while Liu et al. [49] reported opposite findings. This contradiction could be due to variations in the health conditions of the respondents, as healthier subjects were less concerned, and their tendency to travel, as subjects with travel experience were more concerned and anxious about air pollution and its associated health risks [49]. In this study, no significant difference was observed in the awareness of air pollution and its associated health risks between age groups, excluding awareness of major air pollutants, but a significant (*p*<0.05) difference was observed between different age groups for awareness about toxic air pollutants.

The negative impacts of air pollution and its associated health risks can be minimized by increasing the public’s awareness. Internet sources (Baidu/blogs), social apps (Weibo/WeChat), and print or electronic media are useful tools for information transmission, exchange, and flow in China. By using such media, relevant literature and recommended practices against air pollution can be spread [112]. Similarly, informal communications; public conversations; discussions; and exchanging information between relatives, friends, and colleagues can also be vital in creating mass awareness and can positively influence the public’s risk perceptions. Health (doctors/physicians/other health care professionals) and social workers can also be included, as people often adopt health knowledge and practices from them. Based on this study, campaigns, seminars, symposia, and training about air pollution and its associated health risks should be arranged to increase awareness among the public and enable them of self-protection. Regular monitoring of air quality is suggested, as it can lead to fresh, clean, improved, and pollution-free air. During this study, the majority of the respondents reported television, their mobile phone, and the internet as their main sources of knowledge about air pollution and its associated health risks. The same response was reported by Liu et al. [49], however, Bickerstaff and Walker [113] reported that very few people selected media as their primary source of knowledge about air pollution. Although people do not check the air quality index on a regular basis, different reports, commentaries, and coverage about air pollution distributed through the internet, television, newspapers, radio, and magazines are educating people; changing their perception, attitude, and practices towards air pollution; and increasing their awareness of air pollution and its associated adverse health risks.

We attempted to approach as many subjects and to extend the study as broadly as possible, but, as other surveys, we faced some limitations (funding, time, and survey duration). Although the study covered 13 major Chinese cities, there are few rapidly developing cities that should also be surveyed. The current study was embodied to Chinese students only, so involving people other than the students is suggested in future studies. Moreover, for mental and behavioral health, the individual baseline physiological conditions were not adjusted on account of limited time frame, and tight schedule of Chinese students. Furthermore, the administered questionnaire contained closed-ended questions, therefore the responses were “as per asked” by the given questions. Therefore, surveying with an open-ended questionnaire is required to further extend our understanding of the population’s perceptions, attitudes, knowledge, and awareness level, and the problems they face, to determine how their consciousness towards self-protection can be enhanced. These key points must be considered before conducting this study in another area, and these findings should be cautiously applied to another location and population.

## Conclusion

In conclusion, the recruited respondents have suffered from different, adverse physical effects, such as respiratory infections or problems (lung cancer, asthma), and different types of ENT illnesses, due to haze and air pollution. Owing to the current air pollution scenario, almost all of the recruited subjects used face/respiratory masks, some used eyeglasses/goggles to prevent the negative impacts of haze and air pollution, and many ensured that they drank enough water to avoid dehydration and remove toxins. The most severe responses to air pollution were psychologically-associated behavioral problems, indicating a serious threat to mental health, and behavioral vulnerability and variations induced by stress, depression, anxiety, shortened tempers, mood swings, and unpleasant moods. The respondents were aware of major air pollutants, their sources, and the adverse effects of haze and air pollution. Televisions, cell phones, and the internet were the primary sources of knowledge about air pollution and its associated health effects.

Governmental, such as environmental protection agencies, and non-governmental organizations should adopt effective communication styles to educate the public and increase their awareness and understanding of the health risks associated with air pollution at individual, family, and community levels. This will encourage them to adopt preventative measures, and safeguard themselves. This will also maximize the understanding of the public’s risk perceptions, which in turn will not have a negative effect on their behavior. The air quality assessment program should be extended to other parts of China, such as developing cities, which will aid in the acquisition of transparent data regarding air quality parameters in these sites, and will aid the development of proper strategies and plans to improve air quality across China. Coordination between governmental organizations and provincial governments, and their support to the central government is necessary and recommended for developing emission control policies to ensure that future generations do not face these problems.

## Supporting information

S1 File The questionnaire administered/used in the current study. (DOC)

S2 File Supplementary Materials (Tables). Table A. Bonferroni Adjustments to remove family wise error inflation. Table B. Interquartile ranges for the air pollutants across the sampled cities (Oct 2016—March 2016). Table C. Reported gender & city-dependent behavioral and psychological effects of air pollution. Table D. Age-dependent behavioral and psychological effects of air pollution on the recruited respondents. Table E. Gender- and city-dependent adoption of practices to prevent the adverse effects of air pollution. Table F. Age-dependent adoption of practices to prevent the adverse effects of air pollution. Table H. Gender- and city-dependent awareness and perceptions of air pollution. Table G. Age-dependent awareness and perception of air pollution.
(DOC)
