# Supplementary material for: Exposure to air pollution and self-reported effects on Chinese students: A case study of 13 megacities
Source: PLoS One. 2018 Mar 16;13(3):e0194364. doi: 10.1371/journal.pone.0194364 (PMC5856349; doi:10.1371/journal.pone.0194364)
Supplement: S1 File — (DOC) [file pone.0194364.s001.doc]

**SURVEY QUESTIONNAIRE**

**调查问卷**

**A Questionnaire on the Influence of Air Pollution on Human Health and Behavior**

**空气污染和对人类身体健康和行为影响调查**

**Date:**（日期）:______________________ **School/University:**专业/学校：______________

**Age:**（年龄）: ______________________ **Gender** ( M/F):性别（男/女）:______________

**City:**（所在城市）：_________________ **Occupation**:（职业）:______________________

***Direction:*** Please Check (√ ) and rate yourself honestly based on what you actually do given the statements using the following scales: **说明：**请根据您的实际情况在相应位置打( **√ )**，选项说明如下：

| ***Physical/Health effects 健康影响*** | | ***Always总是*** | ***Often经常*** | ***Sometimes 有时*** | ***Rarely偶尔*** | ***Never从不*** |
| --- | --- | --- | --- | --- | --- | --- |
| 1. | In your view, has air pollution ever affected you or your family member or your friend’s health?  您觉得空气污染对您或您的亲友的健康造成了影响吗？ |  |  |  |  |  |
| 2. | How often do you feel sneezing, runny nose, dry throat and eye irritation?  空气质量差时，您经常打喷嚏、流鼻涕、喉咙干和眼睛刺痛吗？ |  |  |  |  |  |
| 3. | How often do you feel shortness of breath/reduced lung functioning?  空气质量差时，您感到呼吸困难或肺功能减弱了吗？ |  |  |  |  |  |
| 4. | How often do you have coughing or wheezing?  空气质量差时，您会经常咳嗽或气喘吗？ |  |  |  |  |  |
| 5. | How often do you get headaches and dizziness?  空气质量差时，您会经常感到头痛或头晕吗？ |  |  |  |  |  |
| 6. | How often do you ever feel reduced energy levels in your body?  空气质量差时，您会经常感觉到身体无力吗？ |  |  |  |  |  |
| 7. | How often do you feel sleep deprivation or sleeping disorder such as insomnia?  空气质量差时，您会经常感到睡眠不足或存在睡眠障碍（如失眠）吗？ |  |  |  |  |  |
| ***Behavioral and psychological effects 行为和心理影响*** | | ***Yes: 是*** | | ***No: 否*** | | |
| 8. | Do you feel sad, depressed or unpleasant at certain times or seasons such as during hazy climate?  在雾霾天气或者雾霾多发的季节，你会经常感觉悲伤、沮丧或者情绪低落吗？ |  | |  | | |
| 9. | Does haze affect your routine exercise i.e.: run or jog faster and for short time?  雾霾天气影响到了你的锻炼吗，比如减少了跑步时间？ |  | |  | | |
| 10. | Does haze affect your routine walking speed, i.e. walking faster？  雾霾天气是否影响你的步行速度，比如走的更快？ |  | |  | | |
| 11. | Do you feel anxiety or frustration?  雾霾天气时，您时常觉得焦虑或失望吗？ |  | |  | | |
| 12. | Do you feel aggressiveness in yourself?  雾霾天气时，您会经常感觉脾气暴躁吗？ |  | |  | | |
| 13. | Do you feel more aggressive during cold days?  天冷时，您会更暴躁吗？ |  | |  | | |
| 14. | Do you feel more aggressive during hot days?  天热时，您会更暴躁吗？ |  | |  | | |
| ***Prevent exposure to hazy contaminants***防止雾霾伤害 | | ***Yes: 是*** | | ***No: 否*** | | |
| 15. | During haze, do you cover your nose and mouth with respiratory masks, when you go out for prolonged periods? (such as N95 masks, are designed to keep out fine particulate matter)  在雾霾环境，如果您长时间在室外，您会戴口罩吗？（比如能有效过滤颗粒污染物的N95口罩） |  | |  | | |
| 16. | During haze, do you wear eye-glasses or goggles to protect your eyes from irritation?  在雾霾环境，您会经常戴防护眼镜以避免眼睛受刺激吗？ |  | |  | | |
| 17 | During haze, do you drink water than usual (because it helps the kidneys flush out any toxins absorbed through the skin and lungs)?  在雾霾环境，您会比平时多喝水吗？（因为多喝水有助于肾脏排出通过皮肤和肺吸入的毒素） |  | |  | | |
| 18. | During haze, do you build up your immunity with foods rich in vitamin C (oranges, guava, strawberries), vitamin E (nuts and seeds) and omega-3 fatty acids (oily [fish](http://www.healthxchange.com.sg/healthyliving/DietandNutrition/Pages/how-to-buy-and-cook-fish-to-boost-your-health.aspx)) or other supplements?  您会经常吃富含维生素C（橙子、石榴、草莓等）、维生素E（坚果和谷物）以及Omega-3脂肪酸（富含油类的鱼）或其他营养品来增强免疫力吗？ |  | |  | | |
| ***People’s awareness and perceptions 意识观念*** | | ***Yes: 是*** | | ***No: 否*** | | |
| 19. | **Do you think smoking should be prohibited in public places and there should be smoking designated areas?**  您觉得公共场所应该禁止吸烟，并设置吸烟专区吗？ |  | |  | | |
| 20. | Do you think respiratory, lung cancer (bronchitis/asthma) and heart diseases related to air pollution are the leading cause of death in China?  您觉得与空气污染相关的呼吸道疾病（气管炎/哮喘）、肺癌以及心脏疾病是中国人的主要死亡原因吗？ |  | |  | | |
| 21. | China’s industrial development grows GDP but lose health because of increase in air pollution. Do you think it’s acceptable and affordable?  中国工业发展促进了GDP的增长却因为空气污染危害了人体健康。您认为这是可以接受且能够承受的吗？ |  | |  | | |
| 22. | Do you know haze exist toxic air pollutants, carbon mono oxide, sulfur dioxide, nitrogen dioxide, particulate matter (PM_2.5_ and PM_10_)?  您知道雾霾中包含有毒污染物么（如一氧化碳、二氧化硫、二氧化氮以及固体颗粒物PM_2.5_和PM_10_）？ |  | |  | | |
| 23. | Which of these factors do you think contributes the greatest amount of haze pollution? Circle the three issues that concern you the most. Please only circle three issues from the list:您认为下列因素哪些是造成雾霾污染的主要原因，请选出三项（仅三项）：(a) Vehicles exhaust汽车尾气 (b) Coal burning 煤炭燃烧 (c) Biomass burning秸秆燃烧(d) Household cooking and heating家庭厨房及供暖 (e) Construction sites dust建筑粉尘(f) Industrial emission工业排放 (g) Smoke of cigarettes个人吸烟 (h) other其他………….. | | | | | |
| 24. | Where have you heard about fog and haze? *Tick as many as you feel apply:*  Television/Radio/Newspaper/Cell-Phone/Internet/Family/Friends/Environmental groups/ or others (*Please write in*):….  您从哪些渠道听说或了解雾霾的？（多选）  电视 / 广播 / 报纸 / 手机 / 因特网 / 家庭 / 朋友 / 环保组织 / 其他 /（请写明_________） | | | | | |
