# Supplementary material for: Exposure to air pollution and self-reported effects on Chinese students: A case study of 13 megacities
Source: PLoS One. 2018 Mar 16;13(3):e0194364. doi: 10.1371/journal.pone.0194364 (PMC5856349; doi:10.1371/journal.pone.0194364)
Supplement: S2 File — Table A. Bonferroni Adjustments to remove family wise error inflation. Table B. Interquartile ranges for the air pollutants across the sampled cities (Oct 2016—March 2016). Table C. Reported gender & city-dependent behavioral and psychological effects of air pollution. Table D. Age-dependent behavioral and psychological effects of air pollution on the recruited respondents. Table E. Gender- and city-dependent adoption of practices to prevent the adverse effects of air pollution. Table F. Age-dependent adoption of practices to prevent the adverse effects of air pollution. Table H. Gender- and city-dependent awareness and perceptions of air pollution. Table G. Age-dependent awareness and perception of air pollution. (DOC) [file pone.0194364.s002.doc]

**S2 File: Supplementary Tables (Table A).** Bonferroni Adjustments to remove family wise error inflation

| **S. No.** | **Bonferroni Correction/ Bonferroni Type Adjustment for α value correction to remove family wise error inflation** |
| --- | --- |
| 1 | Gender Classes (2) * Responses (5 – Always, Often, Sometimes, Rarely, & Never)  Possible Association / Number of Analysis = 2*5 = 10  α_original_ value = 0.05  Binferroni correction = 0.05/10  α_altered_ value = 0.005 |
| 2 | Gender Classes (2) * Responses (2 – Yes, & No)  Possible Association / Number of Analysis = 2*2 = 4  α_original_ value = 0.05  Binferroni correction = 0.05/4  α_altered_ value = 0.0125 |
| 3 | Age Classes (5) * Responses (5 – Always, Often, Sometimes, Rarely, & Never)  Possible Association / Number of Analysis = 5*5 = 25  α_original_ value = 0.05  Binferroni correction = 0.05/25  α_altered_ value = 0.002 |
| 4 | Age Classes (5) * Responses (2 – Yes, & No)  Number of Analysis = 5*2 = 10  α_original_ value = 0.05  Binferroni correction = 0.05/10  α_altered_ value = 0.005 |
| 5 | Cities (13) * Responses (5 – Always, Often, Sometimes, Rarely, & Never)  Number of Analysis = 13*5 = 65  α_original_ value = 0.05  Binferroni correction = 0.05/65  α_altered_ value = 0.001 |
| 6 | Cities (13) * Responses (2 – Yes, & No)  Number of Analysis = 13*2 = 26  α_original_ value = 0.05  Binferroni correction = 0.05/26  α_altered_ value = 0.002 |

**S2 File: Supplementary Tables (Table B)**. Interquartile ranges for the air pollutants across the sampled cities (Oct 2016 - March 2016)

| **Cities** | **Ranges** | **AQI** | **PM 2.5 (μg/m^3^)** | **PM 10 (μg/m^3^)** | **SO_2_ (μg/m^3^)** | **NO_2_ (μg/m^3^)** | **CO (mg/m^3^)** | **O_3_ (μg/m^3^)** |
| --- | --- | --- | --- | --- | --- | --- | --- | --- |
| Nanjing | Q1^a^ | 87.7 | 56.7 | 109.7 | 20.5 | 52.2 | 1.1 | 35.7 |
|  | Q2^b^ | 104.7 | 72.6 | 118.5 | 20.7 | 56.9 | 1.2 | 48.0 |
|  | Q3^c^ | 109.7 | 78.5 | 132.1 | 24.5 | 59.8 | 1.3 | 59.8 |
|  | IQR^d^ | 22.0 | 21.8 | 22.3 | 4.1 | 7.5 | 0.3 | 24.0 |
| Shanghai | Q1 | 77.9 | 52.2 | 70.4 | 15.7 | 47.5 | 0.8 | 43.5 |
|  | Q2 | 79.8 | 55.9 | 75.7 | 17.0 | 52.2 | 0.9 | 59.6 |
|  | Q3 | 97.1 | 69.5 | 84.9 | 22.9 | 56.2 | 1.1 | 77.4 |
|  | IQR | 19.3 | 17.4 | 14.6 | 7.1 | 8.7 | 0.3 | 33.9 |
| Beijing | Q1 | 93.6 | 65.8 | 77.7 | 11.1 | 49.8 | 0.9 | 16.8 |
|  | Q2 | 115.1 | 80.8 | 89.7 | 15.3 | 52.4 | 1.4 | 34.0 |
|  | Q3 | 145.3 | 114.5 | 131.1 | 18.1 | 59.4 | 2.0 | 47.9 |
|  | IQR | 51.8 | 48.7 | 53.3 | 7.0 | 9.6 | 1.0 | 31.0 |
| Hangzhou | Q1 | 76.4 | 52.6 | 84.7 | 13.2 | 48.1 | 0.8 | 24.6 |
|  | Q2 | 90.8 | 64.2 | 103.5 | 14.9 | 53.2 | 0.9 | 42.3 |
|  | Q3 | 102.5 | 74.8 | 116.6 | 16.2 | 58.2 | 1.0 | 62.0 |
|  | IQR | 26.1 | 22.2 | 32.0 | 3.1 | 10.1 | 0.2 | 37.4 |
| XiꞌAn | Q1 | 105.1 | 62.0 | 146.8 | 24.5 | 45.7 | 1.9 | 17.9 |
|  | Q2 | 119.6 | 73.0 | 169.2 | 27.4 | 52.8 | 2.0 | 25.2 |
|  | Q3 | 150.9 | 108.5 | 205.5 | 39.6 | 60.1 | 2.9 | 35.9 |
|  | IQR | 45.8 | 46.6 | 58.7 | 15.1 | 14.3 | 1.0 | 18.1 |
| Chengdu | Q1 | 86.3 | 59.8 | 103.1 | 14.6 | 47.8 | 1.1 | 19.2 |
|  | Q2 | 96.6 | 67.2 | 114.4 | 16.0 | 53.5 | 1.3 | 35.0 |
|  | Q3 | 105.1 | 76.8 | 126.5 | 17.0 | 60.9 | 1.3 | 48.0 |
|  | IQR | 18.8 | 17.0 | 23.4 | 2.4 | 13.2 | 0.3 | 28.8 |
| Baoding | Q1 | 116.5 | 80.6 | 142.4 | 52.4 | 63.4 | 1.4 | 18.9 |
|  | Q2 | 126.2 | 96.1 | 154.2 | 65.5 | 67.5 | 2.3 | 30.8 |
|  | Q3 | 186.5 | 148.4 | 206.5 | 83.7 | 85.5 | 3.5 | 42.8 |
|  | IQR | 70.0 | 67.7 | 64.2 | 31.4 | 22.1 | 2.1 | 23.8 |
| Nanning | Q1 | 60.4 | 39.7 | 63.7 | 12.1 | 31.8 | 1.0 | 28.4 |
|  | Q2 | 64.3 | 43.5 | 71.4 | 12.8 | 35.6 | 1.0 | 33.4 |
|  | Q3 | 79.1 | 51.5 | 85.3 | 15.1 | 36.8 | 1.1 | 46.0 |
|  | IQR | 18.7 | 11.9 | 21.6 | 3.0 | 5.0 | 0.0 | 17.7 |
| Harbin | Q1 | 83.7 | 54.0 | 87.3 | 33.7 | 44.2 | 1.3 | 21.6 |
|  | Q2 | 107.0 | 79.1 | 105.9 | 45.0 | 56.4 | 1.4 | 27.5 |
|  | Q3 | 174.1 | 142.7 | 189.4 | 67.8 | 60.6 | 1.5 | 37.4 |
|  | IQR | 91.4 | 88.7 | 102.0 | 34.1 | 16.4 | 0.2 | 15.8 |
| Lanzhou | Q1 | 91.4 | 45.5 | 112.7 | 16.1 | 44.2 | 1.1 | 27.9 |
|  | Q2 | 94.8 | 59.2 | 125.7 | 24.7 | 51.5 | 1.5 | 37.5 |
|  | Q3 | 104.5 | 61.2 | 138.8 | 33.9 | 57.8 | 1.9 | 48.8 |
|  | IQR | 13.1 | 15.8 | 26.1 | 17.8 | 13.7 | 0.8 | 20.9 |
| Changsha | Q1 | 94.7 | 67.2 | 89.2 | 15.3 | 38.4 | 1.0 | 22.7 |
|  | Q2 | 96.3 | 68.7 | 92.5 | 16.4 | 43.0 | 1.0 | 36.0 |
|  | Q3 | 104.1 | 75.5 | 95.5 | 17.3 | 43.4 | 1.1 | 50.0 |
|  | IQR | 9.5 | 8.2 | 6.4 | 1.9 | 5.0 | 0.9 | 27.3 |
| Shenyang | Q1 | 97.0 | 64.5 | 102.8 | 53.0 | 40.2 | 0.9 | 30.3 |
|  | Q2 | 107.8 | 71.2 | 129.8 | 82.7 | 46.1 | 1.0 | 39.9 |
|  | Q3 | 134.7 | 102.3 | 143.3 | 101.6 | 53.8 | 1.3 | 46.9 |
|  | IQR | 37.7 | 37.8 | 40.5 | 45.6 | 13.6 | 0.4 | 16.5 |
| Wuhan | Q1 | 101.2 | 70.2 | 118.8 | 18.4 | 41.9 | 1.0 | 23.7 |
|  | Q2 | 114.4 | 81.2 | 122.8 | 18.6 | 50.6 | 1.2 | 39.5 |
|  | Q3 | 141.0 | 104.8 | 131.6 | 19.8 | 53.7 | 1.2 | 54.7 |
|  | IQR | 39.8 | 34.6 | 12.8 | 1.4 | 11.8 | 0.2 | 30.9 |

Q1^a^ = 25^th^ percentile, Q2^b^ = 50^th^ percentile (the median), Q3^c^ = 75^th^ percentile, IQR^d^ = Interquartile range = Q_3_ – Q_1_

**S2 File: Supplementary Tables (Table C).** Reported gender & city-dependent behavioral and psychological effects of air pollution

| **Gender** | **Yes** | | **No** | | ***P*-value*** | ***χ*^2^** |
| --- | --- | --- | --- | --- | --- | --- |
|  | ***N*** | **%** | ***n*** | **%** |  |  |
| Feeling sad, depressed and unpleasant during hazy climate | | | | | | |
| Male | 552 | 26.9 | 366 | 17.9 | 0.057^b^ | 3.60 |
| Female | 725 | 35.4 | 404 | 19.7 |  |  |
| Haze effecting daily routine exercise | | | | | | |
| Male | 713 | 34.9 | 203 | 9.9 | 0.728^b^ | 0.12 |
| Female | 886 | 43.3 | 243 | 11.9 |  |  |
| Haze effecting routine exercise speed | | | | | | |
| Male | 581 | 28.4 | 337 | 16.5 | 0.060^b^ | 3,53 |
| Female | 760 | 37.1 | 370 | 18.1 |  |  |
| Anxiety and Depression | | | | | | |
| Male | 495 | 24.2 | 420 | 20.5 | 0.285^b^ | 1.14 |
| Female | 638 | 31.2 | 492 | 24.1 |  |  |
| Aggression/Aggressive Behavior | | | | | | |
| Male | 387 | 18.9 | 530 | 25.9 | 0.122^b^ | 2.39 |
| Female | 515 | 25.2 | 614 | 30.0 |  |  |
| More aggressive in Colder days/season | | | | | | |
| Male | 229 | 11.2 | 689 | 33.6 | **0.014**^b^ | 6.03 |
| Female | 337 | 16.5 | 793 | 38.7 |  |  |
| More aggressive in hotter/warmer days/season | | | | | | |
| Male | 547 | 26.8 | 366 | 17.9 | **0.001**^a^ | 10.39 |
| Female | 753 | 36.9 | 374 | 18.3 |  |  |
| Adverse Behavioral effects reported by the respondents across study sites/cities | | | | | | |
| Nanjing | 1783 | 58.3 | 1275 | 41.7 | **0.000**^a^ | 55.26 |
| Shanghai | 717 | 60.9 | 459 | 39.0 |  |  |
| Beijing | 999 | 55.9 | 786 | 44.0 |  |  |
| Hangzhou | 486 | 57.4 | 361 | 42.6 |  |  |
| XiꞌAn | 713 | 55.9 | 561 | 44.0 |  |  |
| Chengdu | 332 | 62.4 | 200 | 37.6 |  |  |
| Baoding | 561 | 54.2 | 475 | 45.8 |  |  |
| Nanning | 438 | 59.7 | 298 | 40.6 |  |  |
| Harbin | 308 | 51.2 | 294 | 48.8 |  |  |
| Lanzhou | 436 | 51.6 | 409 | 48.4 |  |  |
| Changsha | 855 | 53.3 | 748 | 46.7 |  |  |
| Shenyang | 136 | 64.8 | 74 | 35.2 |  |  |
| Wuhan | 369 | 58.6 | 261 | 41.4 |  |  |
| Total Responses | 8133 | 56.7 | 6201 | 43.3 | 14334 | |

* = bold value represents p-value < 0.05

^a^ = p-value < α_altered_ (Significant after Bonferroni correction); ^b^ = p-value > α_altered_ (Non-Significant after Bonferroni correction)

**S2 File: Supplementary Tables (Table D). Age-dependent behavioral and psychological effects of air pollution on the recrui**ted respondents

| **Age ranges** | **Yes** | | **No** | | ***P*-value*** | ***χ*^2^** |
| --- | --- | --- | --- | --- | --- | --- |
|  | ***n*** | **%** | ***n*** | **%** |  |  |
| Feeling sad, depressed and unpleasant during hazy climate | | | | | | |
| 16-20 | 574 | 58.7 | 404 | 41.3 | **0.003**^a^ | 15.55 |
| 21-25 | 611 | 65.3 | 324 | 34.7 |  |  |
| 26-30 | 71 | 68.3 | 33 | 31.7 |  |  |
| 31-35 | 14 | 87.5 | 2 | 12.5 |  |  |
| ≥ 36 | 8 | 53.3 | 7 | 46.7 |  |  |
| Haze effecting daily routine exercise | | | | | | |
| 16-20 | 742 | 75.9 | 236 | 24.1 | 0.183^b^ | 6.22 |
| 21-25 | 750 | 80.2 | 185 | 19.8 |  |  |
| 26-30 | 85 | 81.7 | 19 | 18.3 |  |  |
| 31-35 | 13 | 81.3 | 3 | 18.8 |  |  |
| ≥ 36 | 12 | 80.0 | 3 | 20.0 |  |  |
| Haze effecting routine exercise speed | | | | | | |
| 16-20 | 626 | 64.0 | 352 | 36.0 | 0.308^b^ | 4.80 |
| 21-25 | 625 | 66.8 | 310 | 33.2 |  |  |
| 26-30 | 67 | 64.4 | 37 | 35.6 |  |  |
| 31-35 | 10 | 62.5 | 6 | 37.5 |  |  |
| ≥ 36 | 13 | 86.7 | 2 | 13.3 |  |  |
| Anxiety and Depression | | | | | | |
| 16-20 | 505 | 51.6 | 473 | 48.4 | **0.034**^b^ | 10.41 |
| 21-25 | 551 | 58.9 | 384 | 41.1 |  |  |
| 26-30 | 59 | 56.7 | 45 | 43.3 |  |  |
| 31-35 | 9 | 56.3 | 7 | 43.8 |  |  |
| ≥ 36 | 8 | 53.3 | 7 | 46.7 |  |  |
| Aggression/Aggressive Behaviour | | | | | | |
| 16-20 | 408 | 41.7 | 570 | 58.3 | 0.229^b^ | 5.62 |
| 21-25 | 436 | 46.6 | 499 | 53.4 |  |  |
| 26-30 | 44 | 42.3 | 60 | 57.7 |  |  |
| 31-35 | 6 | 37.5 | 10 | 62.5 |  |  |
| ≥ 36 | 8 | 53.3 | 7 | 46.7 |  |  |
| More aggressive in Colder days/season | | | | | | |
| 16-20 | 279 | 28.5 | 699 | 71.5 | **0.031**^b^ | 10.63 |
| 21-25 | 265 | 27.1 | 713 | 72.9 |  |  |
| 26-30 | 17 | 16.3 | 87 | 83.7 |  |  |
| 31-35 | 1 | 6.3 | 15 | 93.7 |  |  |
| ≥ 36 | 4 | 26.7 | 11 | 73.3 |  |  |
| More aggressive in hotter/warmer days/season | | | | | | |
| 16-20 | 627 | 64.1 | 351 | 35.9 | 0.143^b^ | 6.85 |
| 21-25 | 596 | 63.7 | 339 | 36.3 |  |  |
| 26-30 | 64 | 61.5 | 40 | 38.5 |  |  |
| 31-35 | 6 | 37.5 | 10 | 62.5 |  |  |
| ≥ 36 | 7 | 46.7 | 8 | 53.3 |  |  |

* = bold value represents p-value < 0.05

^a^ = p-value < α_altered_ (Significant after Bonferroni correction); ^b^ = p-value > α_altered_ (Non-Significant after Bonferroni correction)

**S2 File: Supplementary Tables (Table E). Gender- and city-dependent a**doption of practices to prevent the adverse effects of air pollution

| **Gender** | **Yes** | | **No** | | ***P*-value*** | ***χ*^2^** |
| --- | --- | --- | --- | --- | --- | --- |
|  | ***n*** | **%** | ***n*** | **%** |  |  |
| Use of respiratory mask to cover nose and mouth | | | | | | |
| Male | 561 | 27.4 | 357 | 17.4 | **0.000**^a^ | 38.14 |
| Female | 835 | 40.8 | 295 | 14.4 |  |  |
| Use of glasses/goggles during haze | | | | | | |
| Male | 222 | 10.8 | 696 | 31.0 | **0.033**^b^ | 4.53 |
| Female | 229 | 11.2 | 901 | 44.0 |  |  |
| Drinking enough water | | | | | | |
| Male | 558 | 27.2 | 360 | 17.6 | **0.042**^b^ | 4.12 |
| Female | 736 | 35.9 | 394 | 19.2 |  |  |
| Boost up immunity by eating a rich diet including Vit. C, E, or Omega-3-Fatty acid etc. | | | | | | |
| Male | 569 | 27.8 | 349 | 17.0 | **0.006**^a^ | 7.52 |
| Female | 766 | 37.4 | 364 | 17.8 |  |  |
| Preventive measures adopted by the respondents across study sites/cities | | | | | | |
| Nanjing | 1005 | 57.6 | 739 | 42.4 | **0.000**^a^ | 59.32 |
| Shanghai | 375 | 55.8 | 297 | 44.2 |  |  |
| Beijing | 513 | 50.3 | 507 | 49.7 |  |  |
| Hangzhou | 249 | 51.4 | 235 | 48.6 |  |  |
| XiꞌAn | 401 | 55.1 | 327 | 44.9 |  |  |
| Chengdu | 153 | 50.3 | 151 | 49.7 |  |  |
| Baoding | 302 | 51.0 | 290 | 49.0 |  |  |
| Nanning | 278 | 65.6 | 146 | 34.4 |  |  |
| Harbin | 208 | 60.4 | 136 | 39.6 |  |  |
| Lanzhou | 267 | 55.2 | 217 | 44.8 |  |  |
| Changsha | 488 | 53.3 | 428 | 46.7 |  |  |
| Shenyang | 71 | 59.2 | 49 | 40.8 |  |  |
| Wuhan | 166 | 46.1 | 194 | 53.9 |  |  |
| Total Responses | 4476 | 54.6 | 3716 | 45.4 | 8192 | |

* = bold value represents p-value < 0.05

^a^ = p-value < α_altered_ (Significant after Bonferroni correction); ^b^ = p-value > α_altered_ (Non-Significant after Bonferroni correction)

**S2 File: Supplementary Tables (Table F).** **Age-dependent adoption of practices to prevent the adverse effects of air pollution**

| **Age Ranges** | **Yes** | | **No** | | ***P*-value** | ***χ*^2^** |
| --- | --- | --- | --- | --- | --- | --- |
|  | ***n*** | **%** | ***N*** | **%** |  |  |
| Use of respiratory mask to cover nose and mouth | | | | | | |
| 16-20 | 679 | 69.4 | 299 | 30.6 | 0.306^b^ | 4.82 |
| 21-25 | 629 | 67.3 | 306 | 32.7 |  |  |
| 26-30 | 64 | 61.5 | 40 | 38.5 |  |  |
| 31-35 | 10 | 62.5 | 6 | 37.5 |  |  |
| ≥ 36 | 8 | 53.3 | 7 | 46.7 |  |  |
| Use of glasses/goggles during haze | | | | | | |
| 16-20 | 220 | 22.5 | 758 | 77.5 | 0.389^b^ | 4.13 |
| 21-25 | 196 | 21.0 | 739 | 79.0 |  |  |
| 26-30 | 22 | 21.2 | 82 | 78.8 |  |  |
| 31-35 | 1 | 6.3 | 15 | 93.7 |  |  |
| ≥ 36 | 5 | 33.3 | 10 | 66.7 |  |  |
| Drinking enough water | | | | | | |
| 16-20 | 605 | 61.9 | 373 | 38.1 | 0.109^b^ | 7.55 |
| 21-25 | 593 | 63.4 | 342 | 36.6 |  |  |
| 26-30 | 74 | 71.2 | 30 | 28.8 |  |  |
| 31-35 | 6 | 40.0 | 9 | 60.0 |  |  |
| ≥ 36 | 8 | 53.3 | 7 | 46.7 |  |  |
| Boost up immunity by eating a rich diet including Vit. C, E or Omega-3-Fatty acid etc. | | | | | | |
| 16-20 | 630 | 64.4 | 348 | 35.6 | 0.089^b^ | 8.06 |
| 21-25 | 609 | 65.1 | 326 | 34.9 |  |  |
| 26-30 | 73 | 70.2 | 31 | 29.8 |  |  |
| 31-35 | 11 | 68.8 | 5 | 31.2 |  |  |
| ≥ 36 | 5 | 33.3 | 10 | 66.7 |  |  |

^b^ = p-value > α_altered_ (Non-Significant after Bonferroni correction)

**S2 File: Supplementary Tables (Table G).** Gender- and city-dependent awareness and perceptions of air pollution

| **Gender** | **Yes** | | **No** | | ***P*-value*** | ***χ*^2^** |
| --- | --- | --- | --- | --- | --- | --- |
|  | ***N*** | **%** | ***n*** | **%** |  |  |
| Prevention of smoking in public areas/ There should be smoking designated areas | | | | | | |
| Male | 866 | 42.3 | 52 | 2.5 | **0.000**^a^ | 11.94 |
| Female | 1100 | 53.7 | 30 | 1.5 |  |  |
| Air pollution is linked to respiratory and cardiovascular diseases/disorders | | | | | | |
| Male | 636 | 31.1 | 282 | 13.8 | 0.995^b^ | 0.00 |
| Female | 783 | 38.2 | 347 | 16.9 |  |  |
| China’s growth in GDP affecting the environment, is it acceptable/affordable? | | | | | | |
| Male | 347 | 16.9 | 571 | 27.9 | **0.000**^a^ | 33.22 |
| Female | 293 | 14.3 | 837 | 40.9 |  |  |
| Aware of different toxic substances such as CO, SO_2_, NO_2_, and PM etc. | | | | | | |
| Male | 838 | 40.9 | 80 | 4.0 | 0.362^b^ | 0.83 |
| Female | 1044 | 50.9 | 86 | 4.2 |  |  |
| Awareness level/Perception of the respondents across study sites/cities | | | | | | |
| Nanjing | 1299 | 74.5 | 444 | 25.5 | **0.000^a^** | 40.93 |
| Shanghai | 485 | 72.2 | 187 | 27.8 |  |  |
| Beijing | 708 | 69.4 | 312 | 30.6 |  |  |
| Hangzhou | 361 | 74.6 | 123 | 25.4 |  |  |
| XiꞌAn | 528 | 72.5 | 200 | 27.5 |  |  |
| Chengdu | 215 | 70.7 | 89 | 29.3 |  |  |
| Baoding | 404 | 68.2 | 188 | 31.8 |  |  |
| Nanning | 303 | 71.3 | 122 | 28.7 |  |  |
| Harbin | 213 | 61.9 | 131 | 38.1 |  |  |
| Lanzhou | 346 | 71.5 | 138 | 28.5 |  |  |
| Changsha | 685 | 74.8 | 231 | 25.2 |  |  |
| Shenyang | 83 | 69.3 | 37 | 30.8 |  |  |
| Wuhan | 277 | 76.9 | 83 | 23.1 |  |  |
| Total Responses | 5907 | 72.1 | 2285 | 27.9 | 8192 | |

* = bold value represents p-value < 0.05

^a^ = p-value < α_altered_ (Significant after Bonferroni correction); ^b^ = p-value > α_altered_ (Non-Significant after Bonferroni correction)

**S2 File: Supplementary Tables (Table H).** **Age-dependent awareness and perception of air pollution**

| **Age ranges** | **Yes** | | **No** | | ***P*-value*** | ***χ*^2^** |
| --- | --- | --- | --- | --- | --- | --- |
|  | ***n*** | **%** | ***n*** | **%** |  |  |
| Smoke prevention in public areas/ restricted to smoke designated areas | | | | | | |
| 16-20 | 937 | 95.8 | 41 | 4.2 | 0.517^b^ | 3.25 |
| 21-25 | 891 | 95.3 | 44 | 4.7 |  |  |
| 26-30 | 102 | 98.1 | 2 | 1.9 |  |  |
| 31-35 | 16 | 100.0 | 0 | 0 |  |  |
| ≥ 36 | 15 | 100.0 | 0 | 0 |  |  |
| Air pollution is linked to respiratory and cardiovascular diseases/disorders | | | | | | |
| 16-20 | 669 | 68.4 | 309 | 31.6 | 0.746^b^ | 1.94 |
| 21-25 | 654 | 69.9 | 281 | 28.7 |  |  |
| 26-30 | 70 | 67.3 | 34 | 32.7 |  |  |
| 31-35 | 9 | 56.3 | 7 | 43.8 |  |  |
| ≥ 36 | 10 | 66.7 | 5 | 33.3 |  |  |
| China’s growth in GDP affecting the environment, is it acceptable/affordable? | | | | | | |
| 16-20 | 304 | 31.1 | 674 | 68.9 | 0.384^b^ | 4.16 |
| 21-25 | 298 | 31.9 | 637 | 68.1 |  |  |
| 26-30 | 27 | 25.9 | 77 | 74.0 |  |  |
| 31-35 | 2 | 12.5 | 14 | 87.5 |  |  |
| ≥ 36 | 5 | 33.3 | 10 | 66.7 |  |  |
| Aware of different toxic substances such as CO, SO_2_, NO_2_, and PM etc. | | | | | | |
| 16-20 | 880 | 89.9 | 98 | 10.0 | **0.000**^a^ | 20.21 |
| 21-25 | 874 | 93.5 | 61 | 6.5 |  |  |
| 26-30 | 97 | 93.3 | 7 | 6.7 |  |  |
| 31-35 | 15 | 93.8 | 1 | 6.2 |  |  |
| ≥ 36 | 10 | 66.7 | 5 | 33.3 |  |  |

* = bold value represents p-value < 0.05

^a^ = p-value < α_altered_ (Significant after Bonferroni correction); ^b^ = p-value > α_altered_ (Non-Significant after Bonferroni correction)
